# Assessing the effectiveness of a ‘site visit’ on recruitment rates in a multicentre randomised trial: SWAT-1

**DOI:** 10.1186/1745-6215-16-S2-O10

**Published:** 2015-11-16

**Authors:** Valerie Smith, Mike Clarke, Cecily Begley, Declan Devane

**Affiliations:** 1 Trinity College Dublin, Dublin, Ireland; 2 Queen's University, Belfast, UK; 3 National University of Ireland, Galway, Ireland

## Background

The SWAT (Studies within a Trial) programme, established by the All-Ireland Hub for Trials Methodology Research in collaboration with the Medical Research Council Network of Hubs in the UK and others, is developing methods to resolve uncertainties about trial conduct through embedded research. SWAT-1 provides an initial example.

## Aim

To evaluate the effects of a site visit on recruitment rates in a multi-centre randomised trial.

## Methods

Using the SWAT-1 design, a before-and-after comparison used the date of the site visit as the time point for the intervention. Site A received the site visit. Sites B and C did not receive it and acted as the controls. The primary outcomes were the difference in recruitment in each site from 1 and 3 months pre-intervention to 1 and 3 months post-intervention.

## Results

Recruitment rates increased in Site A post-intervention (17% and 14% percentage point increases at 1 and 3 months, respectively). No differences in recruitment occurred in Site B or in Site C. At 3 months post-intervention, a significant difference was detected in favour of higher recruitment in A compared to B+C (34% versus 25%; odds ratio 1.57, 95% confidence interval 1.09-2.26). These findings suggest that recruitment might increase from 2 participants per week before the site visit to 4-5 per week after it.

## Conclusion

This initial example of a SWAT provides evidence that a site visit increases recruitment rates in a trial. Further SWAT-1s are required to substantiate these findings and to examine the effects in different trials in different settings.

